# The history of circadian rhythm research in Austria

**DOI:** 10.1007/s00508-023-02199-z

**Published:** 2023-05-31

**Authors:** Eva S. Schernhammer, Gerhard Klösch, Isabella Ellinger, Dietmar Winkler, Edda Winkler-Pjrek, Galateja Jordakieva, Kyriaki Papantoniou, Susanne Strohmaier, Bertrand Lell, Franz Waldhauser

**Affiliations:** 1 Department of Epidemiology, Center for Public Health, Medical University of Vienna, Kinderspitalgasse 15, 1090 Vienna, Austria; 2 Channing Division of Network Medicine, Department of Medicine, Brigham and Women’s Hospital and Harvard Medical School, 181 Longwood Avenue, 02115 Boston, MA USA; 3 Complexity Science Hub Vienna, Josefstädter Straße 39, 1080 Vienna, Austria; 4 Department of Neurology, General Hospital Vienna AKH, Medical University of Vienna, 1090 Vienna, Austria; 5 Center for Pathophysiology, Infectiology and Immunology, Institute of Pathophysiology and Allergy Research, 1090 Vienna, Austria; 6 Department of Psychiatry and Psychotherapy, General Hospital Vienna AKH, Medical University of Vienna, 1090 Vienna, Austria; 7 Department of Physical Medicine, Rehabilitation and Occupational Medicine, General Hospital Vienna AKH, Medical University of Vienna, 1090 Vienna, Austria; 8 Center for Medical Statistics, Informatics and Intelligent Systems, Medical University of Vienna, Spitalsgasse 23, 1090 Vienna, Austria; 9 Department for Infectious Diseases and Tropical Medicine, 1st Internal Medicine Department, General Hospital Vienna AKH, Medical University of Vienna, 1090 Vienna, Austria; 10 Centre de Recherches Médicale de Lambaréné, Lambaréné, Gabon; 11 Department for Pediatrics and Adolescent Medicine, General Hospital Vienna AKH, Medical University of Vienna, 1090 Vienna, Austria

**Keywords:** Circadian system, Melatonin, Pineal gland, Vienna, Night shift, Cancer

## Abstract

In view of the recent revival of interest in circadian biology and circadian epidemiology at the Medical University of Vienna, it seems appropriate to highlight the rich and pioneering history of circadian research in Austria. Among the forefathers of circadian research in Vienna are Otto Marburg (1874–1948), who discovered important elements of the pineal gland physiology, Robert Hofstätter (1883–1970), who used pineal gland extract in obstetrics/gynecology, and Paul Engel (1907–1997), who discovered that the pineal gland was controlled by light. More recently, Vera Lapin (1920–2007) showed that surgical removal of the pineal gland increased tumor growth, while Franz Waldhauser (*1946) investigated melatonin in conjunction with night work. Michael Kundi (*1950) and his team conducted among the first studies demonstrating differences in rhythms of night workers and early evidence for health impairments among them. Furthermore, Vienna-born Erhard Haus (1926–2013) pioneered the discovery of the role and importance of melatonin in relation to numerous diseases. This rich pioneering contribution of scientists in Vienna or with roots in Vienna is continued today by a new generation of chronobiologists, epidemiologists and clinicians in Vienna whose new insights contribute to the rapidly developing field of circadian rhythms research. Current topics and contributions relate to the impact of circadian rhythm disruption on health, and the application of chronotherapeutic approaches in clinical and preventive settings.

## Introduction

Vienna boasts a rich history of medical pioneers, including famous physicians such as Rokitansky, Billroth, Semmelweis, Landsteiner, and Freud, to name but a few [1]; however, the field of circadian research is surprisingly “young” in the larger context of medical research. In fact, the term “circadian” was not introduced until 1950 by Franz Halberg (a mentor of Erhard Haus) [2]. Yet, groundbreaking work that paved the way for modern day circadian (sleep) medicine had already been conducted in the decades before, including important contributions by researchers from Vienna. Especially around 1900, Vienna emerged as a global center on various levels including the arts, and science. In this short review, we aim to summarize and highlight the work and history of pioneers of circadian research who were born and/or completed their work in Vienna. We present their biographies and their most important discoveries and contributions in chronological order.

## Otto Marburg (1874–1948)

Otto Marburg was born in Römerstadt, Austria-Hungary (today Rymarov, Czech Republic) and studied medicine at the University of Vienna where he later headed the Neurological Institute for many years. He was compulsorily retired in 1938 and subsequently emigrated to the United States. He died on 13 June 1948 in New York. Several of his family members were murdered at the concentration camp Ghetto Theresienstadt [3].

Otto Marburg was primarily interested in the morphology of the central nervous system, and the physiology of the pineal gland, conducting classical studies of the pineal gland, amongst others. In 1911, he implicated the hormonal nature of the pineal gland [4–6]. He authored a book on sleep disorders and their treatment [7].

On 21 September 1898, Otto Heubner, then the first full professor of pediatrics on German soil and head of the pediatric department at the Charité in Berlin, Germany, presented a 4.5-year-old boy with a pineal tumor and signs of incipient puberty at the 70th meeting of the Leopoldina [8, 9]. Subsequently, several such boys became known and the neurologist Otto Marburg [3, 10], then an assistant at the renowned Neurological Institute of the University of Vienna [11], of which he was later to become director, developed a hypothesis about the function of this organ on the basis of these reports and his histological studies of the pineal gland, which suggested degeneration of the gland after the age of 7 years: “The involution of the pineal gland causes puberty. Let the pineal therefore be a gland whose function must have an inhibitory effect on the genitals” [12] (page 499) [13]. Even then he noticed at that time that, inexplicably, almost exclusively boys were affected by premature sexual maturity, and that animal experiments led to quite contradictory results. Subsequently, a large number of publications emerged, all of which Marburg carefully registered and continuously discussed in review articles [4, 12, 14].

When the National Socialists came to power in Austria in 1938, the Neurological Institute was practically destroyed [11] and pineal gland research in Austria was severely affected: Marburg, like many of his colleagues, had to flee because of their Jewish background [15]. Although he was warmly received in the USA, no further publications on the pineal gland occurred and he died on 13 June 1948 in New York [3]. It was not until 1950 that further publications on the pineal gland appeared in the *Wiener Klinische Wochenschrift*, one by Robert Hofstätter [16] and the other by Paul Engel [17].

Because of the presumed antigonadotropic effect, numerous aqueous pineal extracts came on the market in Europe [18, 19]. The best known of these pineal extracts was probably Epiphysan-Richter, an aqueous extract of 0.1 g of fresh bovine pineal gland, distributed by the Hungarian company Gedion Richter AG [18] and still listed in the Austria Codex in 1996; however, at that time under the name Epiphysan and distributed by Disperga [20].

## Robert Hofstätter (1883–1970)

Robert Matthias Hofstätter was born in Vienna and received his MD from the University of Vienna in 1907. He was a gynecologist and surgeon at the Vienna General Polyclinic (Wiener Allgemeine Poliklinik) and became deputy head of the women’s department of the Children’s Hospital in Vienna in 1938. He died in Vienna on 19 August 1970.

Scientific questions concerning the pineal gland occupied Hofstätter throughout his medical life and work from 1916 to 1960 [21]. He introduced preparations of the pineal gland into gynecological and general medical treatment [21] and researched the importance of the posterior lobe of the pituitary gland for obstetrics [22]. Robert Hofstätter became known in human medicine for his therapeutic experiments with Epiphysan in sexual hyperexcitability [18, 21], premenstrual complaints [23], and also various types of cancer [24, 25]. The predominantly positive effect of pineal gland extracts for sexual hyperexcitability was reportedly confirmed by 21 other authors as early as 1936 [18]. A critical aspect of Hofstätter’s work is that detailed information about the patients was usually lacking and that pineal extracts were often administered simultaneously with various other therapeutic measures, making it difficult to assess their independent effect, a circumstance criticized by Hofstätter himself.

## Paul Engel aka Diego Viga (1907–1997)

Because of his Jewish background Paul Engel fled to South America in 1938, where he adopted the alias “Diego Viga”. Paul Engel was born on 7 June 1907 in Vienna. After receiving his doctorate in 1933 and following a short period of employment at the Vienna General Hospital (II. Chirurgische Klinik, I. Frauenklinik), he became associate professor of endocrinology in Colombia as early as 1938 and was appointed as chair of biology at the Universidad Libre in Bogotá in early 1939 [26]. Because of the emerging civil war and financial problems, he moved to Ecuador in 1948, where he worked as a university professor in Quito, initially teaching twentieth century philosophy at the Universidad Central, and later as a professor of biology at the Faculty of Dentistry in Quito [27]. Engel returned to Europe four times after commencing his exile, his first time following an invitation to the 1st International Congress of Endocrinology in 1959 [28]. While he devoted himself to his scientific work, which he described as his “wife”, he referred to his literary work as his “love” affair [26]. He lived in Quito until his death on 27 August 1997 [27].

His work has anticipated the control of the pineal gland by light. He published more than 20 papers on the pineal gland in German, Spanish and English [29]. These were practically all animal experimental papers and reviews, where he initially concentrated on the connection between the pineal gland, gonadotropic hormone and pituitary growth [30–34]. Engel applied pineal extracts in the context of pubertas praecox and sex organ morphology in animal models, leading to a series of publications in the journals *Wiener Klinischen Wochenschrift *and *Zeitschrift für die gesamte experimentelle Medizin*. Later, his scientific interest shifted from the potential antigonadotropic effect of the pineal gland [30, 33, 35] to its potential significance in tumor growth and cancer [17, 31, 36–40]. In 1936, he contributed a book chapter summarizing the available research and his own experimental findings, conducted partially in collaboration with his colleague Fritz Silberstein, on physiological and pathological functions of the pineal gland, referring to work by Marburg and Hofstätter [41].

## Vera Lapin (1920–2007)

Vera Lapin was born in Prague and received her MD from Prague University in 1945. After several years at the Biomedical and Pharmacological Research Institute and as head of the Academy of Continuing Medical Education in Prague, she fled to Austria in 1968 during the Prague Spring and Warsaw Pact invasion of Czechoslovakia. From 1968 to 1981 Vera Lapin worked at the Institute for Cancer Research at the University of Vienna [42]. She died on 25 August 2007.

Paul Engel’s interest in the pineal gland in relation to cancer was shared by Vera Lapin. She organized the first international conference on “Pineal Gland and Oncogenesis” at the Belvedere in Vienna in 1977 [43]. A key finding of her work was that surgical removal of the pineal gland (pinealectomy) stimulated both the growth of the primary tumor and the formation of metastases thus leading to reduced survival [44, 45].

Much of the further discoveries in pineal gland research and the evolving understanding of the circadian system were subsequently made in the United States. In addition to important discoveries by Aaron Lerner (1920–2007), professor at Yale University who extracted and characterized for the first time the hormone melatonin [46, 47], Lawrance Tamarkin demonstrated the seasonal variation in melatonin production and circadian rhythmicity in Syrian hamsters [48–50] and showed large variation across species. This was the first time that a clearly reproducible physiological function of the pineal gland was established, which also helped to explain the many contradictory results of earlier animal experiments in which neither species nor season played a role. Another Austrian scientist of note, Erhard Haus, became well known for his work in the United States in the 1960s.

## Erhard Haus (1926–2013)

Erhard Haus, who was born on 8 September 1926 to Leo Anton and Marianne Haus in Vienna, Austria, was the grandson of Anton Haus, the last Grand Admiral of the Austrian-Hungarian Empire. He received his degree in internal medicine from the Leopold Franzens University Innsbruck Faculty of Medicine in 1951 and did his residency in internal medicine in Paris and Vienna, before immigrating to the United States in 1958. He earned both a medical degree in pathology and a PhD in pathology/physiology from the University of Minnesota, where he held an academic appointment in the department of pathology for more than 40 years. In 1966, he joined the prestigious department of anatomic and clinical pathology at the Saint Paul Ramsey Medical Center (now Regions Hospital) in Saint Paul, Minnesota, where he served as chief of the department and residency training program for nearly 35 years [51].

Erhard Haus’ work was devoted to medical chronobiology (endogenous biological rhythms). As principal investigator or collaborator he conducted more than 520 national and international research projects dealing with circadian rhythm desynchronization (jet lag, shift work), chronobiology of the endocrine and cardiovascular systems, cancer and cancer risk, and nutrition and metabolic disorders/obesity. His pioneering research furthered the concepts of chrononutrition, chronopharmacology, chronotherapeutics, chronotoxicology, and diagnostic chronobiology (time-qualified reference values). He lectured at numerous national and international conferences and courses on diverse aspects of medical chronobiology, edited 3 books, and authored/coauthored more than 65 book chapters, 260 journal articles, and 300 abstracts. Additionally, he founded and was president of the American Association for Medical Chronobiology and Chronotherapeutics and served as a chair of the World Health Organization’s Section on Mechanisms of the International Agency for the Research on Cancer, Working Group on the Evaluation of Carcinogenic Risks to Humans in Painters, Fire Fighters, and Shift Workers, which addressed the risk for breast, prostate, and other cancers of shift and night workers exposed to light at night [52].

## Franz Waldhauser (*1946)

Franz Waldhauser is a pediatric endocrinologist at the University Hospital for Pediatrics and Adolescent Medicine in Vienna, a specialist in pediatrics and adolescent medicine with a specialty in childhood hormonal disorders and was deputy head of the Department of General Pediatrics at the General Hospital in Vienna until 2003. His scientific work refers to more than 200 publications.

Franz Waldhauser was fascinated by Marburg’s hypothesis in the late 1970s because it offered an explanation for children with idiopathic pubertas praecox. During his 18-month research visit at Richard J. Wurtman’s laboratory of neuroendocrine regulation (MIT Boston), one of the first functioning radioimmunoassays (RIA) for melatonin was developed and substantially higher nocturnal blood melatonin levels were detected in prepubertal children than later in life [53]. Although this finding was consistent with Marburg’s hypothesis, the first boy with pubertal symptoms in human chorionic gonadotropin (HCG)-producing pineal tumors was reported in 1981 [54]. This and similar observation offered an alternative explanation for the association of pineal tumors and pubertal signs in boys and rendered Marburg’s hypothesis obsolete: HCG stimulates the testosterone-producing Leydig cells in the infantile testis and the testosterone thus produced leads to signs of puberty, better pseudopuberty, in boys.

## Michael Kundi (*1950)

Michael Kundi, who was born in Vienna on 31 May 1950, was the head of the Institute of Environmental Health of the Medical University of Vienna from 2004 until 2017. Before that, he was head of the staff unit for epidemiology and methodology at the Institute of Environmental Hygiene (1991–1996), and head of the Department of Occupational and Social Hygiene from 1996. Kundi studied psychology, medicine and mathematics at the University of Vienna, graduating in 1979. In 1989, he received his habilitation in epidemiology and occupational health from the Medical Faculty of the University of Vienna.

A team around Michael Kundi from the Medical University of Vienna, including Margit Koller, Manfred Haider, and Renate Cervinka, investigated different aspects of shift work. In a small but unique work among night workers, the authors measured melatonin and cortisol in saliva and presented early evidence for marked differences in rhythms among night workers as well as for the importance of an early morning light stimulus to keep the circadian system in sync [55]. In 1989, Kundi had also been among the first to provide suggestive evidence for early indicators of health impairment among night workers from the oil industry in Austria based on indicators of destabilization over time [56].

Early precursors to Kundi’s work were some experimental shift work studies, including one by Heinrich Goldstein (1891—unknown; unregistered death during the Nazi regime), a native of Vienna, who published in 1913 on the effect of night work on body temperature in humans [57]. Several studies at that time investigated whether a sudden change in the light-dark cycle, such as occurs during night work, results in a complete reversal of the temperature cycle (i.e. complete entrainment), with the ultimate goal of finding shift schedules/rotations that are most beneficial/least detrimental to human physiology. This and other related works are summarized in a review by Knauth and Rutenfranz (1976) on “Circadian rhythm and body temperature—re-entrainment at shift change”; [58] Goldstein’s work contributed to the then unresolved question whether night work reverses body temperature; Goldstein’s work did not find such a reversal [57]. Given the incomplete understanding of the circadian system at the time and the widely divergent study conditions involved, it is not surprising that consistent results were not obtained.

## Conclusion

More than 100 years of research on circadian rhythms have set the ground for the recognition of the importance of circadian rhythms in medicine and public health. Austrian researchers have been integral to circadian research from the early days of discoveries of the circadian system and its anatomical description, to our current, deeper understanding of its molecular structure and importance for healthy aging. In its infancy, this research was interrupted in Austria by World War II, but individual groups have continued to make selective, albeit disjointed, contributions to 1) the discovery and anatomical description of the pineal gland, 2) the function of the hormones secreted by the pineal gland, 3) the impact of melatonin on tumor growths in mammals, and 4) early studies of shift workers. More recently, a multidisciplinary research group has formed at the Medical University of Vienna, including the authors of this article. Given the rich contribution of pioneering scientists with roots in Vienna, it may not be surprising that the tradition continues today with this new generation of chronobiologists, epidemiologists and clinicians currently based in Vienna contributing new insights into the rapidly evolving field of circadian rhythms. Together, they hope to make a more concerted effort towards personalized interventions and further delineation of disease-relevant aspects of the circadian system. Some examples of topics of current studies at the Medical University of Vienna include acute and chronic health effects of night work, circadian disruption due to nighttime light exposure, mistimed daily exposure (e.g., meal timing) and associations with chronic diseases in the general population, as well as the evaluation of application of circadian concepts such as the time of drug administration or vaccination on patients’ outcomes. Their success is evidenced by several hundred publications on the topic, most of these in circadian epidemiology, as well as funded research projects to identify individual factors that contribute to disease risk when circadian rhythms are disrupted, and to intervene.

While the field is rapidly evolving, we hope that the rich Viennese research tradition will inspire young researchers to become future researchers in the field of circadian rhythms and practitioners of circadian medicine.
